# Axenfeld Rieger Syndrome Presenting with Open Angle Glaucoma in an Adult Patient: A Case Report

**DOI:** 10.31729/jnma.8951

**Published:** 2025-04-30

**Authors:** Madhu Thapa, Pragati Gautam, Sanjeeta Sitaula

**Affiliations:** 1 B.P. Koirala Lions Center For Ophthalmic Studies, Department of Ophthalmology, Maharajgunj Medical Campus, Maharajgunj, Kathmandu, Nepal

**Keywords:** *anterior segment dysgenesis*, *Axenfeld Rieger Syndrome*, *glaucoma*

## Abstract

Axenfeld Rieger Syndrome is autosomal dominant genetic condition, which can present with various ocular and non-ocular findings. Anterior segment dysgenesis is the most common ocular finding leading to glaucoma. Non-ocular findings include craniofacial abnormalities, cardiac, dental as well as neurological problems. Mutations in PITX2 and FOXC1 genes have been associated with this condition. FOXC1 mutation causes more ocular findings where as PITX2 mutation has been linked with ocular as well as craniofacial abnormalities. Racial or gender predilection has not been suggested by any literature. Vision loss in these patients is mainly due to uncontrolled glaucoma which needs to be diagnosed and treated urgently.

## INTRODUCTION

Axenfeld Rieger syndrome (ARS) presents with ocular developmental delay which has autosomal dominant mode of inheritance with no sex predilection.^1^ This is also called as anterior chamber cleavage syndrome and mesodermal dysgenesis of cornea and iris. This condition is seen in approximately 1 out of 200000 live births.^2,3^

Clinical features of ARS include posterior embryotoxon in cornea, which is anteriorly placed Schwalbe's line. Occasionally they might present with megalocornea, microcornea and central corneal opacity. On evaluation of angle, there would be thick, prominent Schwalbe's line with iris stands over the angle which is usually open. Iris will have significant stromal atrophy with polycoria, corectopia and at times ectopian uveae.^4^

Patients with ARS are at approximately a 50% risk for the development of glaucoma. Glaucoma may present in childhood or early adulthood. Various other findings like strabismus, limbal dermoid, corneal pannus, persistent pupillary membrane, chorioretinal coloboma and retinal detachment can also be present. Among systemic features, dental abnormalities are very common. Dental features such as hypodontia, microdontia, oligodentia, enamel hypoplasia, conicalshaped teeth, shortened roots and delayed eruption. The facial abnormalities include maxillary hypoplasia, hypertelorism, telecanthus, broad, flat nasal bridge.

In addition, patients may have hypospadias, anal stenosis, pituitary abnormalities, growth retardation, and cardiac valvular abnormalities.^4^

## Case

A 18-years-old female presented to our outpatient department with history of blurring of vision for distance for few years and bitemporal headache for few weeks. She gave history of irregular use of topical beta blocker, Timolol (0.5%) eye drops twice daily for last 2 years. She was a short statured lady with hypodontia and microdontia with under developed facial bones. Her Best Corrected Visual Acquity (BCVA) was 6/24 in her right eye and 6/6 in her left eye. On examination, she had microcormea with white-to-white corneal diameter of 8.5 mm. Posterior embryotoxon seen all around the limbus in right eye. The pupil is peaked inferiorly with iridocorneal adhesion at 6 o'clock position. Relative Afferent Pupillary Defect (RAPD) was noted her right eye. Left eye corneal diameter was normal with prominent posterior embryotoxon with prominent iris strands in peripheral iris and angle. On Retinal evaluation, retina looked normal in both eyes but there was advanced cupping of 0.9:1 vertically and 0.7:1 horizontally with significant notch in the inferiorly in right eye but no glaucomatous change in left eye. Goldman applanation tonometry showed Intra Ocular Pressure (IOP) of 40 mmHg and 18 mmHg respectively in right eye and left eye. Goniscopy showed open angle with prominent Schwalbe's line with iris strands scattered over the angle in both the eyes. Her Humphry visual field showed advanced visual field defect (MD value of -12db) in her right eye along with significant Retinal Nerve Fiber Layer (RNFL) thinning in Optical Coherence Tomography (OCT). There were no changes in visual field and OCT in her left eye. She was diagnosed as a case of open angle glaucoma secondary to anterior segment dysgenesis in her right eye.

Medical consultation reveled her to be ARS without cardiac or other systemic association.

Combination of α-adrenergic agonist, Brimonidine (0.2%) and β-blocker, Timolol (0.5%) was prescribed. After two weeks her IOP was still 28 mmHg in right eye and 22 mmHg in her left eye. Then, Prostaglandin analogue, Brimatoprost (0.03%) was added in her both eyes.

Intra Ocular Pressure (IOP) did not control with addition of antiglaucoma drops in her right eye and she was advised for right eye filtration surgery and to continue same drug in her left eye. After the Trabeculectomy her IOP was under control ranging from 12-15 mmHg over the period of one year. Left eye IOP was controlled on Brimatoprost(0.03%) without progressive glaucomatous changes.

**Figure 1:**
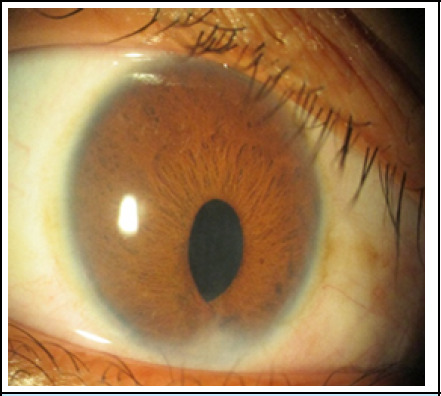
Right eye showing microcornea with peaked pupil due to iridocorneal adhesion at 6 o'clock.

**Figure 2:**
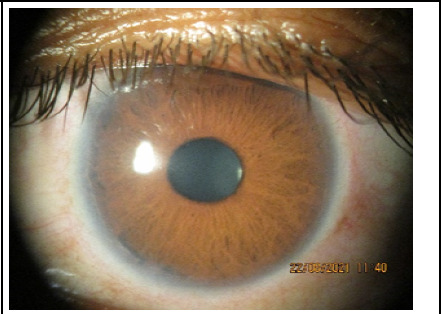
Prominent posterior embryotoxon at limbus in the left eye.

**Figure 3:**
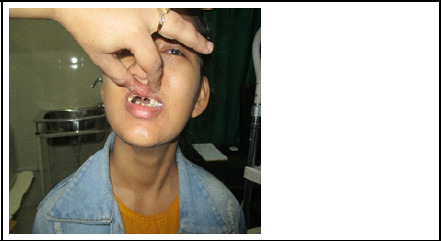
Patient showing Microdentia, oligodentia.

**Figure 4:**
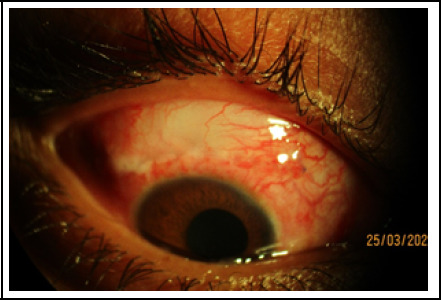
Diffuse bleb after Trabeculectomy.

## DISCUSSION

Impaired neural crest cell migration and differentiation during embryonic development are considered important in the pathogenesis of ARS.^2^ Sixty percent of cases have been linked to mutations in the transcription factors PITX2 (4q25), FOXc1 (6p25) and unidentified genes on 13q14, 16q24 and chromosome 11.^5^ Axenfeld Rieger syndrome (ARS) is the congenital anterior segment anomaly which can lead to glaucomatous optic nerve damage in around half of the patients. ^2,3,6^

The Glaucoma can be identified in early childhood but sometimes they can present in adult only. Glaucoma associated with this kind of anomaly is refractory to medical management and this needs surgical management in a significant number of cases. Trabeculectomy with anti-fibrotic agents are found to have a good long-term result. Glaucoma drainage device is also found to have a good IOP control. Sometimes patient might need multiple glaucoma surgeries in case the IOP is not controlled with primary surgery.^7^

Although a good long-term IOP control can often be achieved in childhood glaucoma, the visual acuity remains below the normal range in most cases despite close follow-up.^8^

## CONCLUSION

Axenfeld Rieger syndrome can present with glaucoma in adult as well. Glaucoma associated with this condition is often refractory to medical treatment and might need surgical intervention to control the IOP and to prevent further optic nerve damage.
